# Analgesic evaluation of ultrasound-guided Pericapsular Nerve Group (PENG) block for emergency hip surgery in fragile patients: a case series

**DOI:** 10.1186/s42836-019-0018-0

**Published:** 2019-12-30

**Authors:** Tommaso Pagano, Fulvio Scarpato, Gianmaria Chicone, Domenico Carbone, Carlo Blandina Bussemi, Francesco Albano, Fabio Ruotolo

**Affiliations:** Anesthesia and Intensive Care Unit, Umberto I Hospital, Nocera Inferiore- ASL, Salerno, Italy

**Keywords:** Analgesia, Novel ultrasound-guided block, Hip surgery, Fragile patient - elderly

## Abstract

**Background:**

Emergency hip surgery is common especially in elderly patients. Very often we are faced with elderly and fragile patients with several comorbidities. In these cases a careful pain control is crucial to reduce length of stay, costs, postoperative complications and mortality. Currently the Fascia Iliaca Block (FIB) and the Femoral Nerve Block (FNB) are the main techniques used for this purpose.

**Cases presentation:**

Recently, a new method has been described under ultrasound-guidance, the Pericapsular Nerve Group (PENG) block. In this case series we try to point out the importance of this novel, safe and effective ultrasound-guided locoregional analgesic technique as an alternative to FIB or FNB based on our clinical experience.

**Conclusion:**

In this case series the PENG block has been proved to be safe and effective, but more and larger-sized studies are needed to better assess the method in future before it becomes an established analgesic technique for hip surgery.

## Background

Hip fracture represents a frequent orthopedic emergency in elderly patients, and it is associated with significant mortality and morbidity [1]. Surgical reduction and fixation are the definitive treatment in most patients [2]. To relieve the pain around hip capsule remains the most important analgesic target for this type of surgery. Effective perioperative analgesia that minimizes the need for opioids and related adverse effects (respiratory depression, nausea, vomiting and delirium mainly), and improves health-related quality of life, is essential in this population of patients [3, 4]. Some techniques, such as Femoral Nerve Block (FNB) and Fascia Iliaca Block (FIB), are popular regional analgesic strategies, because of their opioid-sparing effects [5–7]. The analgesic effect size of these blockades is only moderate and literature showed that the obturator nerve (ON) is often not adequately covered [8]. ON, accessory obturator nerve (AON) and femoral nerve (FN) innervate the anterior hip capsule as reported in previous anatomical studies [9–11] and it is the most richly innervated section of the joint, suggesting these nerves might serve as main targets for hip analgesia. A recent anatomical study by Short et al. [12–14] confirmed this, but also found that the AON and the FN play a greater role in the anterior hip innervation than previously reported. Moreover, this study also put in evidence important landmarks for those articular branches. The high articular branches from FN and AON are consistently found between the anterior inferior iliac spine (AIIS) and the iliopubic eminence (IPE), whereas the ON is located close to the infero-medial acetabulum. By using these information, an innovative ultrasound-guided technique for blockade of these articular branches to the hip, the Pericapsular Nerve Group (PENG) block, has been recently developed and described by Girón-Arango et al. [15]. The PENG blockade can block both FN and AON. In this case series we described the technique, its effectiveness and feasibility in 6 elderly and fragile patients scheduled for emergency hip surgery.

## Case series

**Fig. 1:**
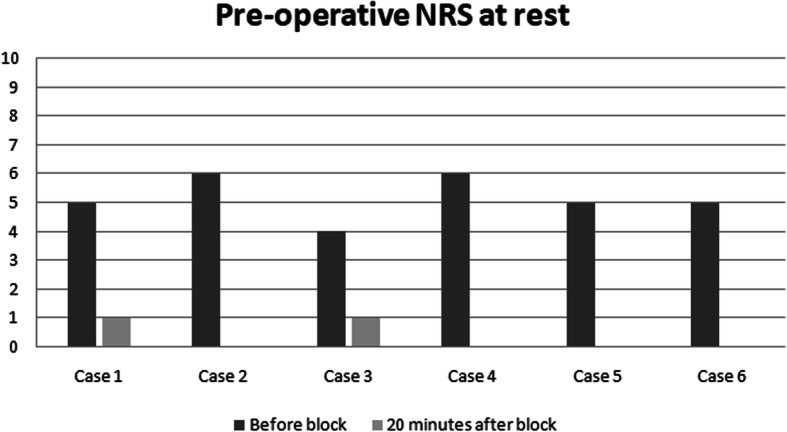
Pre-operative NRS at rest

**Fig. 2:**
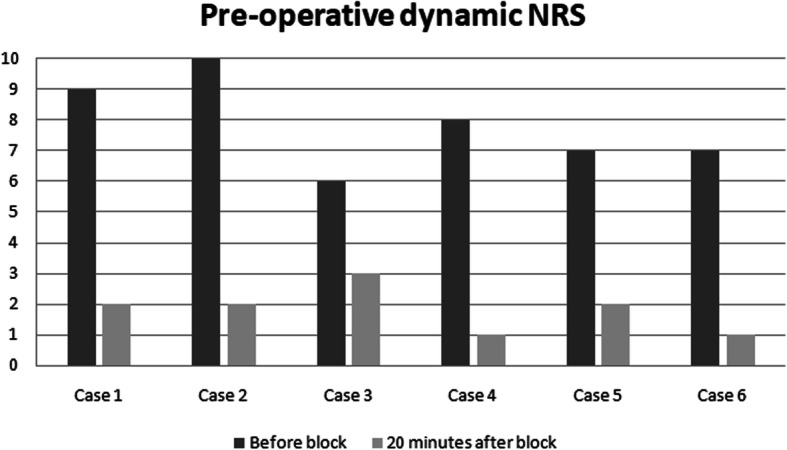
Pre-operative dynamic NRS

The PENG block has been performed in 6 elderly and fragile patients with hip fracture, after written informed consent was obtained. Pain scores, at rest and dynamic, with a straight leg raise of the affected limb to 15 degrees, were assessed before and 20 minutes after blockade by using Numerical Rating Scale (NRS). Before the block was performed, severe pain was reported at rest by all patients despite of intravenous ketorolac at 30 mg (Fig. 1). Twenty minutes after blockade, all patients were able to comply and reported significantly reduced dynamic pain in terms of scores compared with baseline (Fig. 2). No quadriceps weakness was noticed in any patient.

**Fig. 3:**
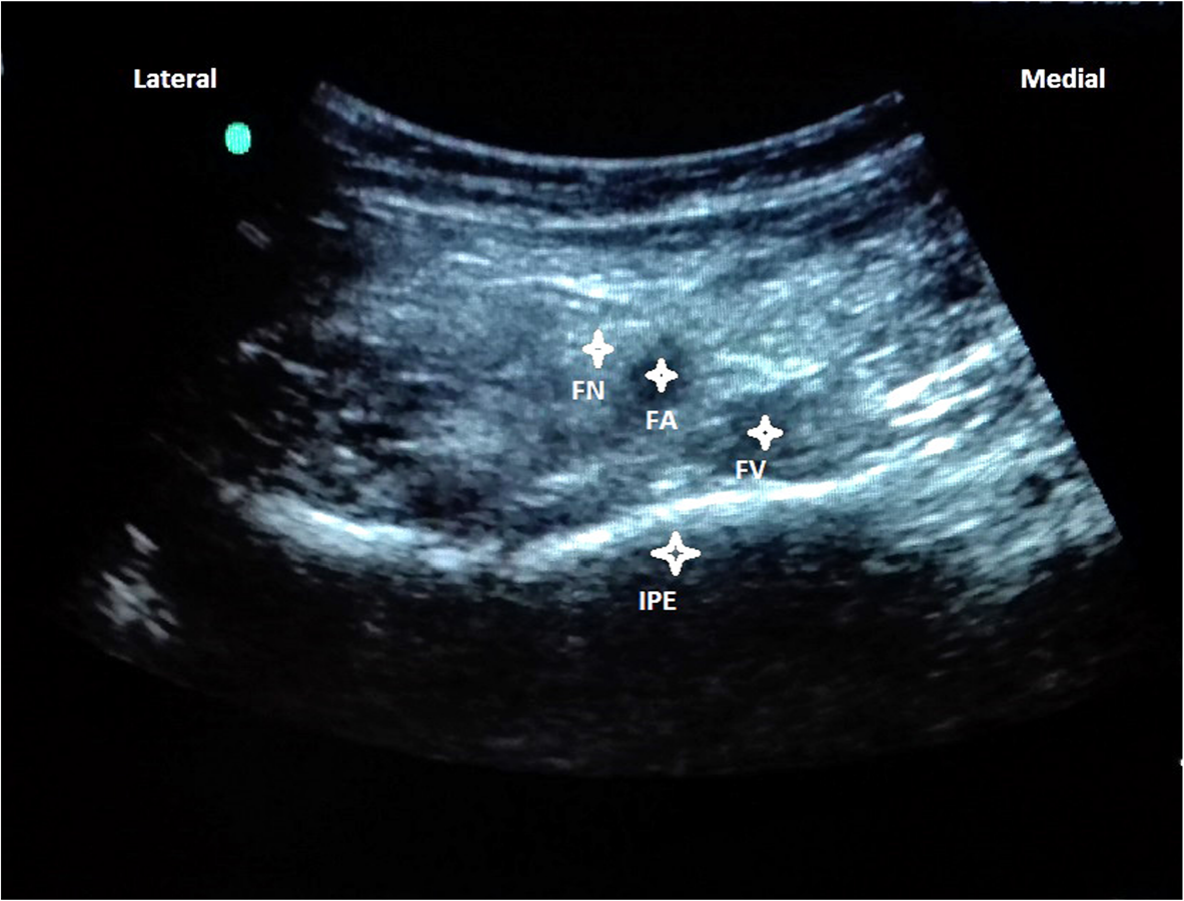
Femoral nerve (FN), artery (FA), vein (FV), Iliopubic eminence (IPE)

**Fig. 4:**
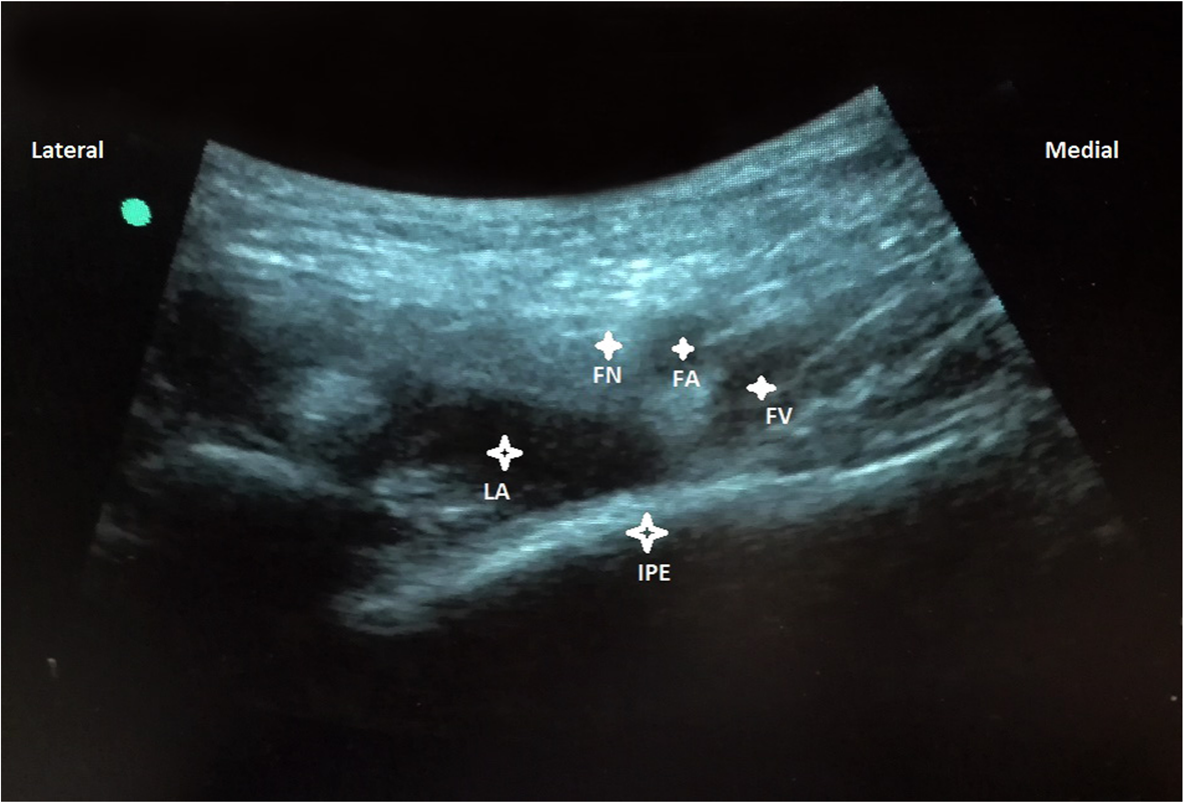
Femoral nerve (FN), artery (FA), vein (FV), Iliopubic eminence (IPE), Local anesthetic mixture (LA)

With the patients in supine position, a convex low-frequency (2–5 MHz) ultrasound probe was placed on a transverse plane over the AIIS and then it was aligned with the pubic ramus by rotating the probe counterclockwise at approximately 45 degrees. On this ultrasonographic view, the IPE, femoral nerve and vessels were clearly observed (Fig. 3). So with in-plane approach a 20-gauge and 100-mm needle was inserted from lateral to medial to place the tip in the musculofascial plane between the pubic ramus posteriorly and the psoas tendon anteriorly. Following negative resistence and aspiration tests, the local anesthetic solution composed of a mixture of mepivacaine 1% and ropivacaine 0,5% was slowly injected while observing for adequate fluid spread for a total volume of 20 mL (Fig. 4).

All blockades were performed after obtaining an adequate venous access, with vital parameters observed and sterility criteria followed. Intralipid 20% was promptly available in operating room, and other proactive measures, such as emergency drugs, tracheal intubation equipment and oxygen source, were ready. We have respected the same contraindications concerning other types of ultrasound-guided peripheral blockades.

**Fig. 5:**
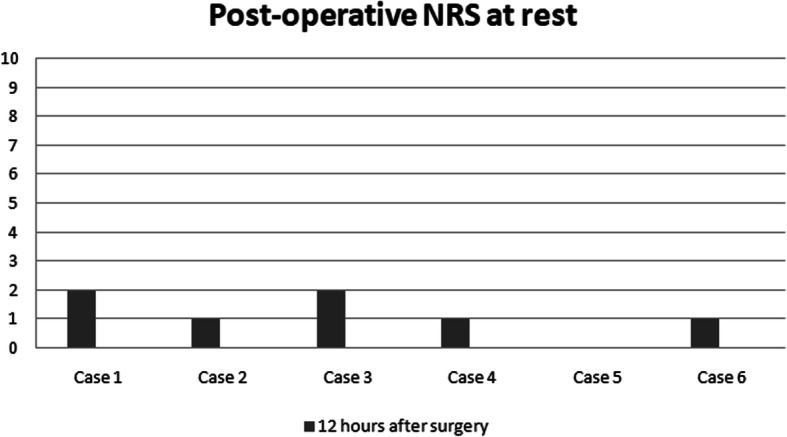
Post-operativa NRS at rest

**Fig. 6:**
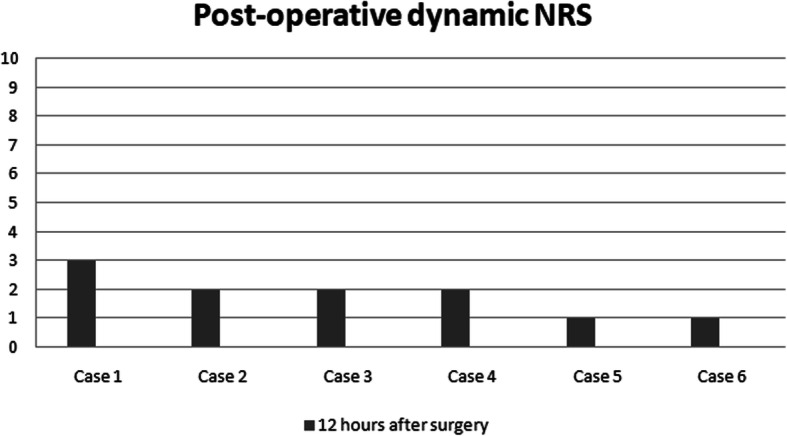
Post-operative dynamic NRS

Thirty minutes after PENG block, patients received spinal anesthesia with 12.5 mg levobupivacaine 0,5%. Twelve hours after surgery, NRS was evaluated again (Figs. 5 and 6) and neither opioids nor non-steroidal anti-inflammatory drugs (NSAIDs) were required during this period. The Table 1 shows demographic and epidemiological data of the patients.

**Table 1:** Case series

Case	ASA	Age, y	Gender	Hip pathology	Type of surgery
1	III	76	F	Intertrochanteric fracture	DHS fixation
2	III	80	F	Subcapital fracture	IMN
3	IV	88	M	Subcapital fracture	IMN
4	III	82	M	Intertrochanteric fracture	DHS fixation
5	III	72	F	Subcapital fracture	IMN
6	IV	91	F	Intertrochanteric fracture	DHS fixation

*ASA* American Society of Anesthesiologists, *DHS* dynamic hip screw, *IMN* intramedullary nail, *F* female, *M* male

## Discussion and conclusions

In patients with hip fractures regional analgesic blocks are often useful.

A recent Cochrane review on nerve blockades in hip fractures, which included FNB and FIB, has shown high-quality evidence supporting a reduction in dynamic pain within 30 min of blockade. In this review the effect size was − 3,4 points on a scale from 0 to 10 [8]. The cephalad spread of local anesthetic in FNB and FIB has been examined with magnetic resonance imagining. The ON is not widely covered. More importantly, the cephalad spread is unlikely to extend beyond the L5 level. Recent anatomical studies demonstrated that the articular branches from the FN, before innervating the hip capsule, enter the iliacus muscle at the L4–L5 levels and course deep to the psoas muscle and tendon between the AIIS and IPE. The AON courses deep to the medial aspect of psoas muscle around the L5 level, then it courses deep to the psoas around IPE to enter the anteromedial joint capsule [12, 13]. In contrast, the targets of the regional blockades described in our case series were the articular branches of AON and FN between AIIS and IPE. We are not able to affirm if the local anesthetic solution would spread medially enough to reach the plane between the pectineus and obturator externus muscles (subpectineal plane, SPP) where the articular branches of ON can be found. The SPP has been recently described by Nielsen et al. [14] as a target point for ON and its articular branches. Given the proximity of the SPP, it is conceivable that the local anesthetic may have spread to this plane. Anyway, dye injection studies are necessary to confirm this.

The median reduction of pain in our case series was 4,83 points in preoperative NRS at rest, and 6 points in dynamic state. Therefore, NRS was evaluated again 12 h after surgery, both in dynamic state and at rest. Interestingly, the patients in our case series presented different hip pathologies (intertrochanteric and subcapital fractures), and all of them reported significant preoperative pain relief and satisfactory postoperative analgesia. In addition, given that our technique targeted only the sensory branches, there was a potential motor-sparing effect compared with both the FIB and the FNB.

Nowadays there are no randomized controlled trials or other large-scale studies in literature regarding the PENG block. This is only a small case series and there are many limitations inherent to this type of study, such as danger of overinterpretation, lack of ability to generalize, publication bias and the retrospective nature of the design. But this type of publications also has some merits, such as the detection of novelties and generation of stimuli and hypotheses. There is room for improving the effect size of analgesia compared to the FIB and FNB, as discussed before. This case series shows a very impressive effect of this new blockade on the dynamic pain score and a good postoperative analgesia. This case series may help to consider a new approach of nerve blockades for patients with hip fracture with the better understanding of the anatomy for hip innervation and the planes where the nerves to the hip innervation run. We need more cadaveric studies, dye injection studies to confirm the spread of local anesthetics and randomized controlled trial to establish its efficacy, safety, and advantages over other regional analgesic techniques. Furthermore, in the near future, studies concerning optimal volume and type of local anestethics, any adjuvant drugs and particular populations, such as obese patients, will also be needed.

## Data Availability

All data generated or analysed during this study are included in this published article.

## Abbreviations

AIIS: Anterior inferior iliac spine
AON: Accessory obturator nerve
FIB: Fascia Iliaca Block
FN: Femoral nerve
FNB: Femoral Nerve Block
IPE: Iliopubic eminence
NRS: Numerical Rating Scale
NSAIDs: Non-steroidal anti-inflammatory drugs
ON: Obturator nerve
PENG: Pericapsular Nerve Group
SPP: Subpectineal plane
